# Elements of Anatomy

**Published:** 1845-01

**Authors:** 


Art. IV.—*Elements of Anatomy, intended as a Text-book for Students.* By Alex. J. Lizars, m.d., Professor of Anatomy in Marischall College, Aberdeen. Part III, *containing the Organs of Nutrition and Generation.—Edinburgh*, 1845. 12mo.

This is the concluding portion of a text-book of which we gave a very favorable report on the appearance of the first part. (Brit. and For. Med. Rev. vol. IX, p. 539.) The small work preserves its character to the close, and we can conscientiously recommend it to the student as an excellent manual which, in the words of the author, "will enable him to follow lectures on anatomy with every possible facility, and will supply him, at the termination of his studies, with the readiest means of reviewing this branch of science within the narrowest limits."

